# Changes in functional neuroimaging measures as novices gain proficiency on the fundamentals of laparoscopic surgery suturing task

**DOI:** 10.1117/1.NPh.10.2.023521

**Published:** 2023-05-05

**Authors:** Yaoyu Fu, Pushpinder Walia, Steven D. Schwaitzberg, Xavier Intes, Suvranu De, Anirban Dutta, Lora Cavuoto

**Affiliations:** a University at Buffalo, Department of Industrial and Systems Engineering, Buffalo, New York, United States; b University at Buffalo, Jacobs School of Medicine and Biomedical Sciences, Department of Surgery, Buffalo, New York, United States; c Rensselaer Polytechnic Institute, Center for Modeling, Simulation, & Imaging in Medicine (CeMSIM), Troy, New York, United States; d University at Buffalo, Department of Biomedical Engineering, Buffalo, New York, United States

**Keywords:** functional near infrared spectroscopy, surgical skills assessment, suturing

## Abstract

**Significance:**

As trainees practice fundamental surgical skills, they typically rely on performance measures such as time and errors, which are limited in their sensitivity.

**Aim:**

The goal of our study was to evaluate the use of portable neuroimaging measures to map the neural processes associated with learning basic surgical skills.

**Approach:**

Twenty-one subjects completed 15 sessions of training on the fundamentals of laparoscopic surgery (FLS) suture with intracorporeal knot-tying task in a box trainer. Functional near infrared spectroscopy data were recorded using an optode montage that covered the prefrontal and sensorimotor brain areas throughout the task. Average oxy-hemoglobin (HbO) changes were determined for repetitions performed during the first week of training compared with the third week of training. Statistical differences between the time periods were evaluated using a general linear model of the HbO changes.

**Results:**

Average performance scores across task repetitions increased significantly from the first day to the last day of training (p<0.01). During the first day of training, there was significant lateral prefrontal cortex (PFC) activation. On the final day, significant activation was observed in the PFC, as well as the sensorimotor areas. When comparing the two periods, significant differences in activation (p<0.05) were found for the right medial PFC and the right inferior parietal gyrus. While gaining proficiency, trainees activated the perception-action cycle to build a perceptual model and then apply the model to improve task execution.

**Conclusions:**

Learners engaged the sensorimotor areas more substantially as they developed skill on the FLS suturing task. These findings are consistent with findings for the FLS pattern cutting task and contribute to the development of objective metrics for skill evaluation.

## 1 Introduction

As trainees practice fundamental surgical skills, they typically rely on performance measures, such as time and errors, to determine improvement. For example, each manual skill of the fundamentals of laparoscopic surgery (FLS) curriculum, the standard for basic surgical skills training of US-based surgeons, is evaluated based on a score combining the completion time and task-specific errors. However, these measures may be limited in their sensitivity and utility for providing feedback to trainees to improve performance. In addition, there is often a mismatch between these measures that are the focus of simulation training and the expectations of experienced surgeons in the clinical environment.^1^ As a result, a recent survey determined that many still require additional training in laparoscopic suturing, including needle positioning and bimanual coordination, after completing residency.^2^ Thus, there has been a push in surgical skills training to support the development of objective metrics to improve assessment and training.

Three main categories of objective measurement modalities have emerged in the surgical skills training literature: video-based assessment, eye tracking, and neuroimaging. Experts and novices have been differentiated based on tool movement, including parameters such as movement speed and path length, which is typically extracted from videos of task performance.^3^ Experts exhibit smoother task execution, with fewer pauses and less jerkiness.^3^ Although computer vision techniques have enabled semiautomated tool identification and skill assessment from videos, video-based assessment is often limited by the quality of the recordings and the need for large datasets to build reliable models relevant to a specific task.^4^ Recent studies on the use of eye tracking and gaze-based metrics for medical skills assessment have shown differentiation between novice and expert providers. For example, Richstone et al.^5^ and Liu et al.^6^ identified increased focus during surgical tasks by expert surgeons, as indicated through a higher fixation rate compared to novices. However, eye-tracking data collection is particularly sensitive to lighting conditions, and assessing dynamic areas of interest for head-mounted eye trackers requires frame-by-frame analysis of video components, which is burdensome for long duration tasks in naturalistic settings. Underlying both tool movement and eye tracking is the central nervous system control of the motor system. Thus, measurement of the neural correlates, captured using portable neuroimaging techniques, for motor learning and surgical skills proficiency has been of increasing interest in the literature.

Over the past 15 years, there has been a growing interest in the use of functional near infrared spectroscopy (fNIRS) as a neuroimaging approach for assessing surgical skill. For both open surgical knot tying and laparoscopic knot tying, significantly elevated hemodynamic response in the prefrontal cortex (PFC) was observed for surgical trainees compared to experts.^7^^–^^10^ These studies have been consistent with the motor learning literature, where the PFC is typically associated with attention, sensorimotor association, and working memory,^11^ particularly during the cognitive phase of learning.^12^ As individuals transition to the autonomous phase and the attentional demand drops, PFC attenuation is expected and has been reported in multiple studies.^7^^,^^13^^,^^14^ However, Shetty et al.^15^ reported that despite similar performance scores between experts and novices following some training, there were significant differences in PFC activation, which indicates that the trainees still experienced cognitive load on the task, and may not have attained the automatic stage of learning. These findings support the use of neuroimaging-based metrics, as the typical proficiency standards were not able to differentiate the insufficiency of learning.^7^ In addition, they support the need for developing a detailed understanding of how the neuroimaging-based metrics change as individuals transition from a novice to a proficient but not quite expert, skill level.

Although a few of the previous studies that have used fNIRS have considered laparoscopic surgical skills, there have been key limitations to the analyses. First, almost all previous studies have been limited to the PFC. For complex bimanual tasks, such as the FLS intracorporeal suturing with knot-tying task (FLS suturing), other brain regions are expected to be recruited. One recent study comparing experts and novices for the FLS precision cutting task found significant differences for the primary motor cortex (M1) and supplementary motor area (SMA).^16^^,^^17^ M1 is involved with fine motor control and complex motor task performance, and the SMA is associated with bimanual coordination. Thus, these areas are relevant for understanding the neural processes for skill acquisition on this complex bimanual task. Second, only one recent study has looked at an extended training period (e.g., 2 weeks) of sufficient length to reach performance scores comparable to expert levels.^15^ Others have considered shorter training periods, such as 45 min^13^ and 4 days,^14^ which are insufficient to effectively learn a complex task, such as intracorporeal suturing. These training studies were also restricted to PFC assessment. Based on the finding from Shetty et al.^15^ where PFC attenuation did not accompany proficient-level performance scores, it is critical to integrate this longer training period with the expanded cortical region coverage to better evaluate the learning process. In another training study that did look at additional cortical regions (including PFC, SMA, and M1), Nemani et al.^16^ focused only on cortical activation when performing a transfer task for precision cutting for groups that trained on either a virtual or physical simulator. The emphasis for their study was on the ability to use machine learning to classify training modality based on activation during task transfer, rather than understanding the differences in activation across a wider range of regions, and they only considered group sizes of five to seven participants. Third, the previous studies have not considered the corresponding cortical activation when comparing those who performed better during the training period versus those who did not learn as well.

Given the limitations of prior studies in terms of a focus on comparing expert versus novice performance, short training durations when considering skill acquisition, small sample sizes, and limited cortical region coverage, the goal of this study was to evaluate the use of portable neuroimaging measures to map the neural processes associated with learning fundamental surgical skills, namely suturing with intracorporeal knot tying, toward the goal of developing further objective measures of skill development. It was hypothesized that the acquisition of suturing skill would be associated with changes in cortical activation pattern, with an expected transition from areas associated with attention and working memory to those associated with motor task execution.

## 2 Methods

### 2.1 Participants

Twenty-one medical and undergraduate students in healthcare-related programs participated in this study. The demographics for those participants that completed the study are shown in Table 1. The study was approved by the University at Buffalo Institutional Review Board and all participants were provided written informed consent at the beginning of their first session.

**Table 1:** Participants demographics.

Age, average (range)	23.71 (18 to 36)
Gender, female:male	13:8
Hand dominance, left:right	0:21
Med students, yes:no	7:14

### 2.2 Experimental Design

**Fig. 1:**
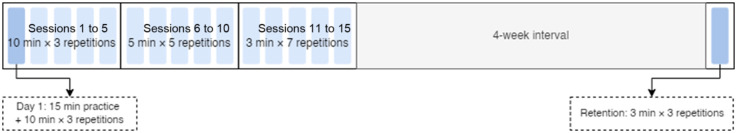
Experiment procedures.

Each participant completed 15 sessions of training (75 repetitions of the task) over a 3-week period and 1 follow-up session after 4 weeks of no training (Fig. 1).

The task training was structured in a block design pattern. Each session started with a 2-min resting block where the participants were instructed to relax and minimize their movement. Then they began the first task practice block and performed the task to completion, or until the maximum practice time was reached. At the end of the practice block, they were instructed to stop, and a new 2-min resting block started. If the participant finished the task before the maximum practice time, they were instructed to relax and wait until the practice block finished. Each practice block lasted 10 min for sessions 1 to 5, 5 min for sessions 6 to 10, and 3 min for sessions 11 to 15, to reflect the faster pace of task completion with learning. Participants completed three task repetitions per session during the first five sessions, five repetitions per session during the second five sessions, and seven repetitions per session for the last five sessions (for 75 total repetitions, ∼6.5  h of training). Based on previous studies of laparoscopic suturing training and the FLS curriculum standards, this was expected to provide a sufficient number of replications for participants to reach proficiency.^18^ Four weeks after the last training session, the participants returned for another session to measure their skill retention. During this session, they performed three repetitions of the training task.

### 2.3 FLS Suturing Task and Task Performance

**Fig. 2:**
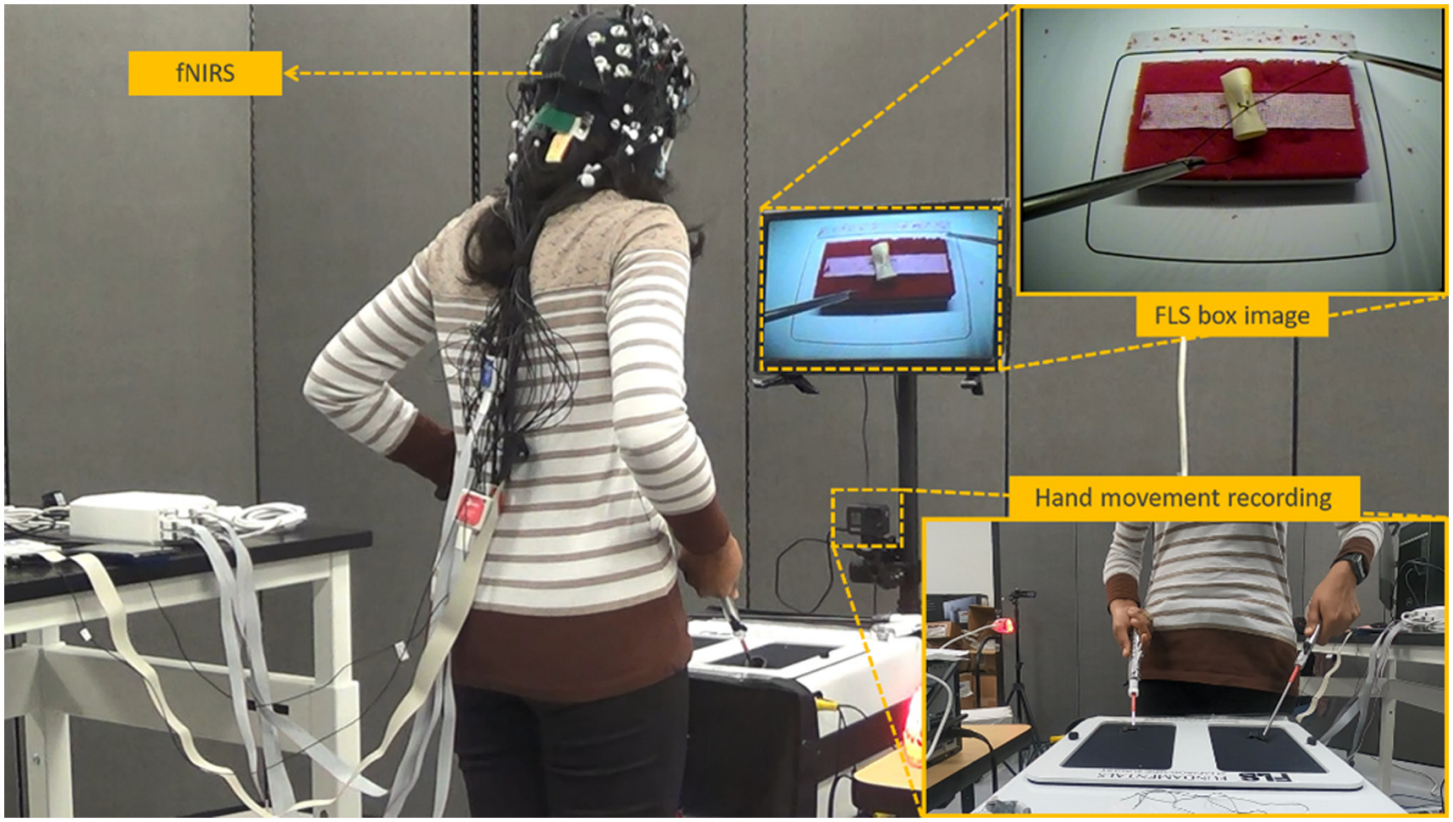
Experimental setup.

The task in this study was the FLS suture with intracorporeal knot-tying task performed in a standard FLS box trainer (as shown in Fig. 2). At the beginning of the first session, the participant watched a tutorial video of the suturing task^19^ then was given 15 min to practice the task with verbal instruction from the researcher until they remembered the steps of the task. The task performance was measured following the FLS guidelines,^19^ and the performance score was based on the task completion time and errors (deviations to the targets, gap in incision, and knot security).^18^

### 2.4 Data Acquisition and Preprocessing

**Fig. 3:**
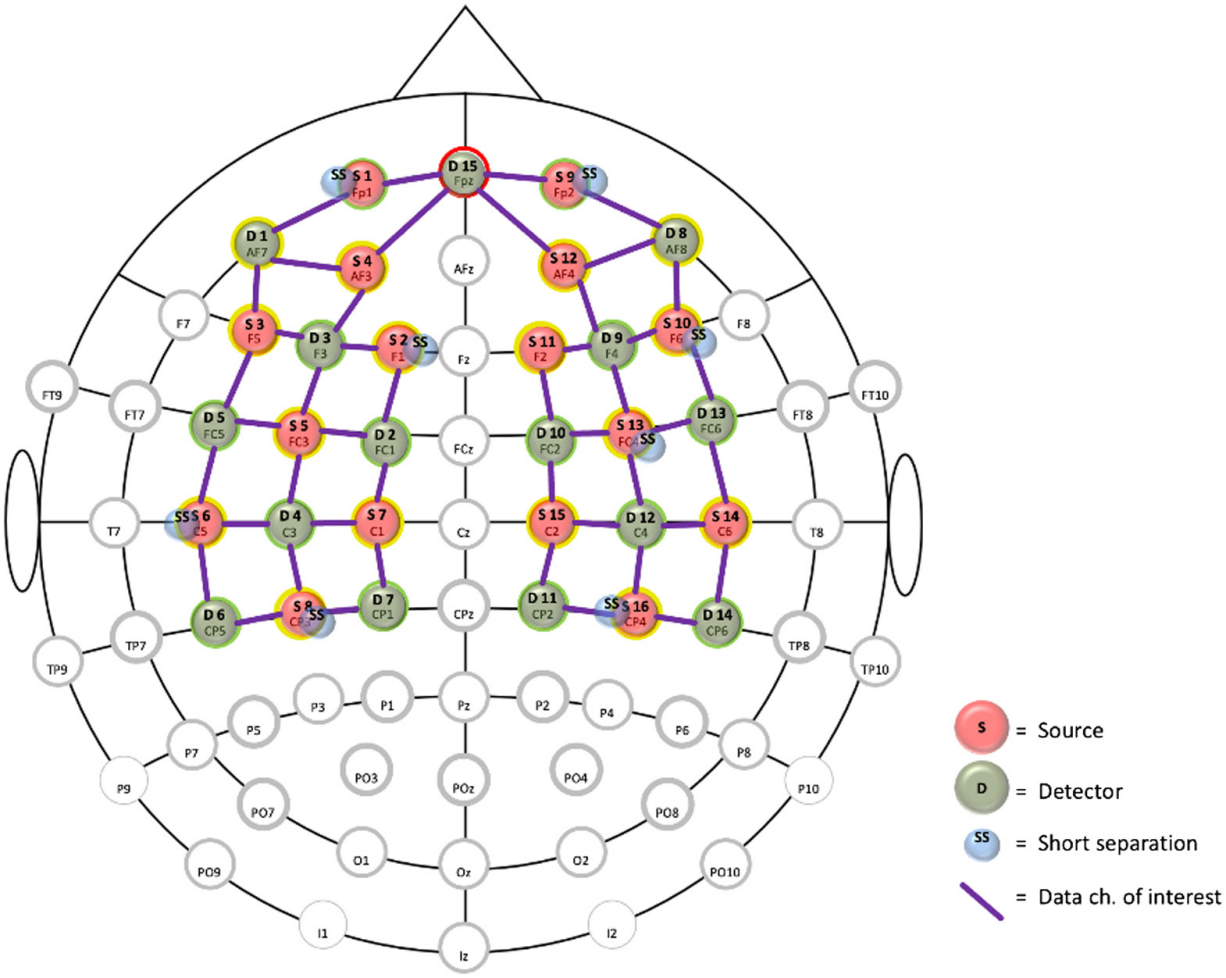
fNIRS montage covering the prefrontal, sensorimotor, and motor areas.

fNIRS data were recorded throughout the session with the NIRSport2 (NIRx Medizintechnik GmbH, Germany) with a sampling rate of 5 Hz. The system emitted light at 760 and 850 nm. Participants wore a cap (Fig. 2) containing 16 sources, 16 detectors, and 8 short-distance detectors that covered the prefrontal, sensorimotor, and motor brain areas (Fig. 3).

The fNIRS data was processed using the open-source HOMER3 package (https://github.com/BUNPC/Homer3). First, the detected light intensity (760 and 850 nm) was converted to optical density. Then, motion artifacts were removed using the Savitzky–Golay method (default parameters) in HOMER3.^20^ Then the optical density was bandpass filtered in the neurovascular coupling band, 0.01 to 0.1 Hz, and converted to oxygenated hemoglobin (HbO) and deoxygenated hemoglobin (HbR) concentration changes using the modified Beer–Lambert law.^21^ The partial pathlength factors of 1.0 and 1.0 were used to get micromolar (μM) concentration units for HbO and HbR, respectively. The short-separation channels (<15  mm, optimum distance ∼8  mm for adults^21^) were regressed from the long-separation channels (see data channels of interest in Fig. 3) to remove the signal changes in the extracerebral layer. This was performed in the general linear model (GLM) in HOMER3 that was used to calculate the hemodynamic response function (HRF) at each source-detector pair using ordinary least squares. The HRF model used consecutive sequence of Gaussian basis functions with a standard deviation of 0.5 and means separated by 0.5 sec for a time range of −2 to 60 s.

### 2.5 Statistical Analysis

Task performance for each repetition during the training and retention sessions. A repeated measures analysis of variance was used to compare the suturing task performance across day 1, day 15, and retention. Post hoc analyses with a Bonferroni adjustment were performed for pairwise comparisons.

The learning curve across all 15 sessions for each participant was assessed with the cumulative summation test for learning curve (LC-CUSUM).^22^ This approach provides a visual representation of whether and when a learner reaches a defined performance level (proficiency). A negative direction of the LC-CUSUM line indicates success and a positive direction indicates failure. When the learner’s score crosses the defined decision threshold, they are considered to have learned the task.^22^ The acceptable failure rate p0 was set at 10% and the unacceptable failure rate p1 was set at 25%. The criterion score was defined based on previous studies.^22^^,^^23^ If the participant’s performance was better than the criterion score, the task was considered a success, and when below the criterion score, it was considered a failure. The success and failure sample weight were −0.0792 and +0.398, respectively. The decision threshold was set at −3.7.

For statistical analysis, we used HbO changes due to higher amplitude and higher signal-to-noise-ratio^23^ since we addressed the confounding physiological interferences with short-separation regression. The HbO changes were determined from the averaged HRF for repetitions performed on each day of training and were compared by adapting HOMER3 function (“hmrS_CalcPvalue”) for day 1 and day 15 against baseline, and the two time periods were also compared against each other. Here, the statistical differences were evaluated using only a GLM using the first 20 s of the HRF.^24^ Channel-level differences were compared using a paired t-test. For analysis of the effect of level of learning on the neuroimaging results, participants were divided into two groups based on their final day performance, those who were above (good learners) versus those who were below the mean (weak learners). Independent samples t-test was used to evaluate differences between subgroups. For all statistical analyses, significance was set at p<0.05. HbR, although more robust to systemic changes, lacks statistical power^23^ and is presented in the Supplementary Material.

## 3 Results

### 3.1 Task Performance and Learning Curves

**Fig. 4:**
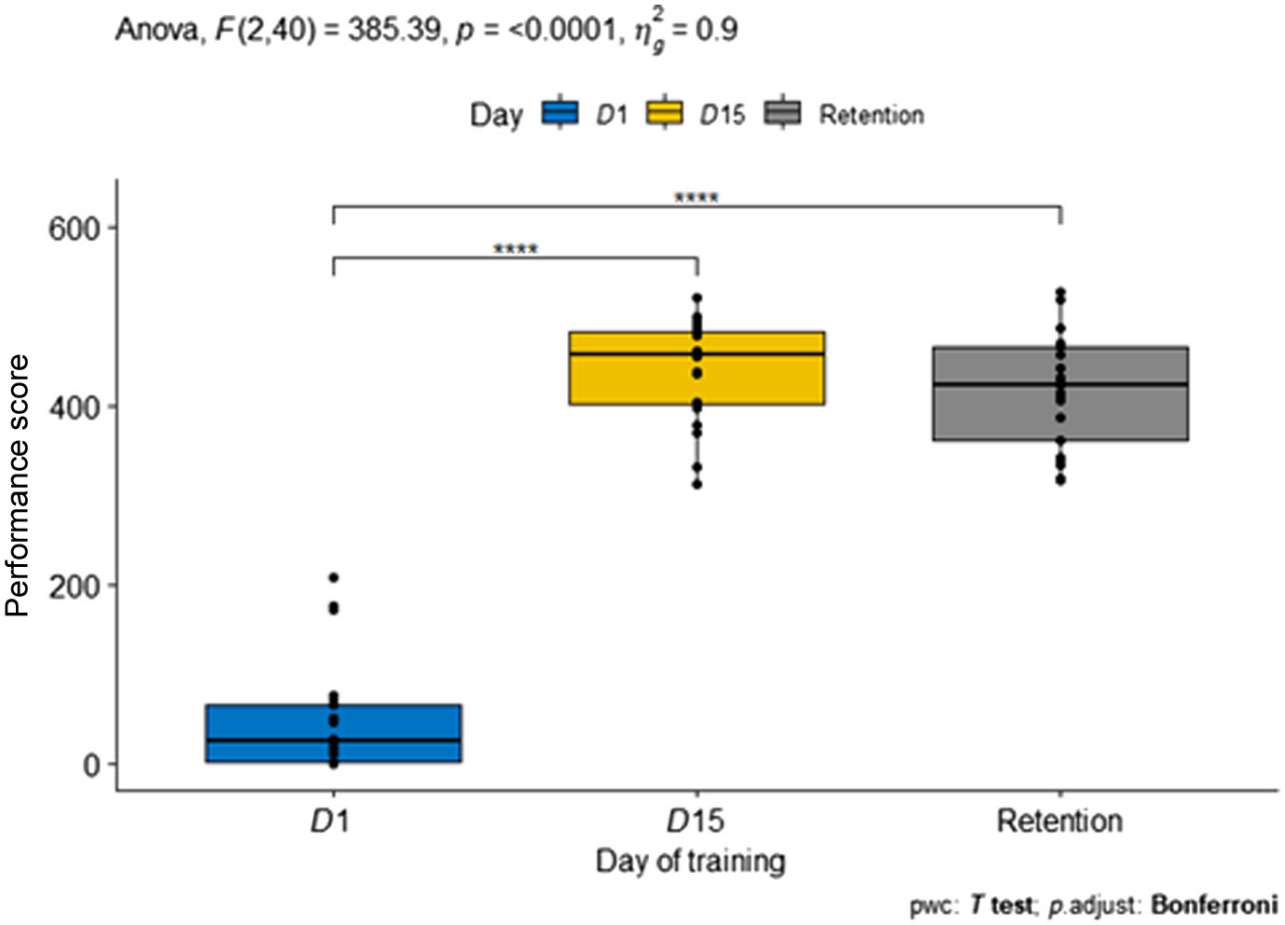
Statistical comparison between first, last, and retention day performance.

As shown in Fig. 4, there was a significant main effect of time period on performance. As all participants were novices to laparoscopic suturing, performance on the first day was low. Only two participants achieved day 1 performance <150. The day 1 score was significantly different than the day 15 and retention session scores. The average performance on day 1 was 48.6±62.4, and it was increased significantly to 439.9±57.6 on day 15. The performance ability was retained after 4 weeks (418.6±66.4), and day 15 and the retention session were statistically equivalent. Average task time was ∼483±103  s for day 1 (see Table 2). By day 15, the average task time for the final three repetitions was ∼114±25  s. Deviation from the marked dots improved from an average 1.47±1.1  mm to 0.56±0.59  mm.

**Table 2:** Performance score comparisons.

	Time	Deviation	Score
Day 1	483.22 ±102.78	1.47 ± 1.10	48.59 ± 62.42
Day 15 (last three repetitions)	113.81 ± 24.56	0.56 ±0.59	439.92 ± 57.55
Retention	126.07 ± 31.05	1.00 ± 0.99	418.59 ± 66.41

**Fig. 5:**
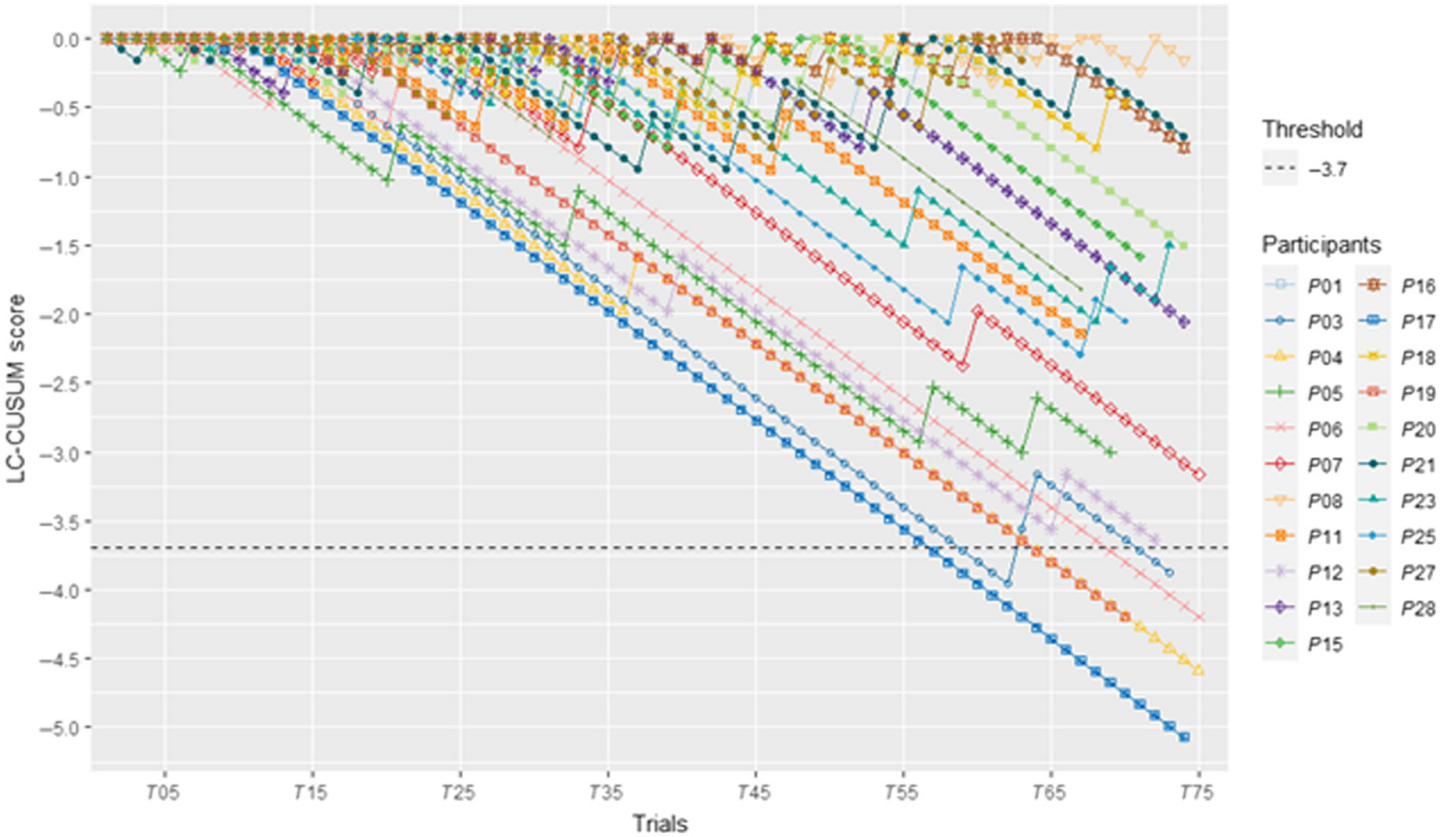
LC-CUSUM lines for the 21 participants.

Five trainees (P03, P04, P06, P17, and P19; see Fig. 5) crossed the decision threshold before reaching repetition 75. Three other trainees (P05, P07, and P12) reached an LC-CUSUM score below −3. Four participants (P01, P08, P7, and P18) had weak LC-CUSUM performance and finished with scores between 0 and −0.5. Across the 75 repetitions, the average task time across participants was 191.7 (39.1) s, and the average penalty score was 72.5 (33.1).

### 3.2 Neuroimaging Analysis

**Fig. 6:**
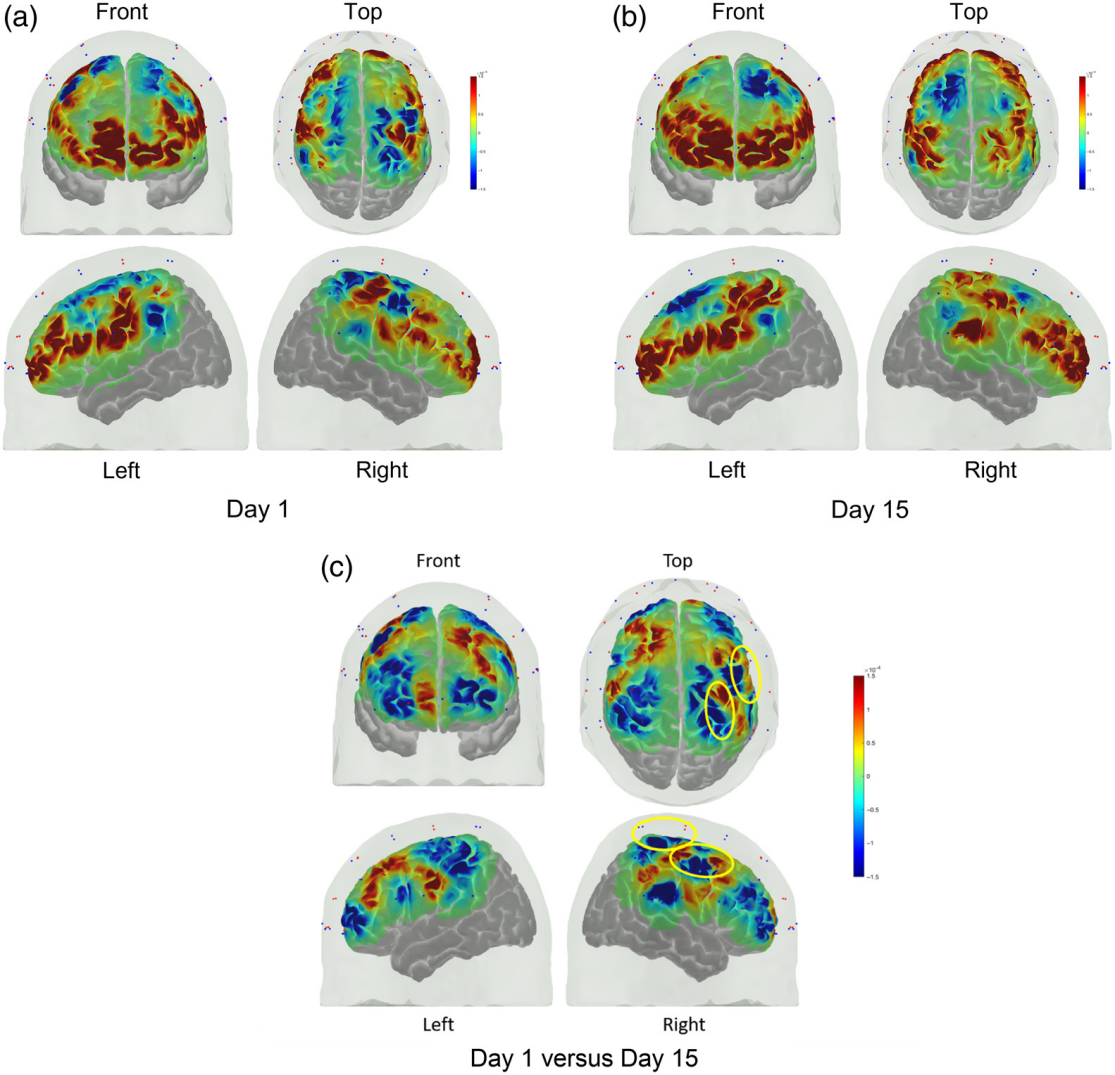
(a) Average activation at the group level on the first day of training and (b) last day of training and (c) the contrast between days. The significant areas for the contrast are circled in yellow.

Across repetitions on the first day of training, there was a significant (p<0.05) change in concentration from baseline for the channels shown in Fig. 6. A full listing of the statistical analysis results for HbO and HbR are included in the Supplemental Material in Tables S1 and S2. The underlying areas corresponding to these channels include the left ventrolateral PFC (inferior frontal gyrus), bilateral dorsolateral PFC, and the bilateral rolandic cortex. On the last training day, significant activation from baseline was found on the bilateral dorsolateral PFC, bilateral ventrolateral PFC, and the area covering the superior temporal gyrus and inferior parietal lobule. When comparing the two periods, significant differences in activation were found for the right precentral gyrus and the right superior parietal lobule. In both cases, the activation was higher for day 15 than for day 1.

In further analyzing activation for day 15, the mean final day performance of 439 was used to divide the participants. Twelve participants scored above this value (range of 454 to 521) and nine participants scored below (range of 312 to 437). Those in the high-performing group averaged higher activation than the below mean group for the left anterior cingulate, insula, dorsolateral PFC, inferior frontal gyrus pars opercularis, and inferior parietal lobule, as well as the right inferior frontal gyrus pars opercularis and postcentral gyrus. The results are from the group level statistical analysis of the HRF (0 to 20 s).

## 4 Discussion

In this study, we evaluated the use of portable neuroimaging measures to map the neural processes associated with learning the FLS suturing with intracorporeal knot-tying task. It was hypothesized that the acquisition of suturing skill would be related to a transition in cortical activation. Overall, the study showed a rightward shift in the PFC activation from the first to the last day of the training. We postulate that the first day of training engaged implicit learning of the complex FLS task environment while the last day of training engaged explicit learning where the subject had knowledge of the task environment. Here, the hemispheric laterality in the engagement of PFC is important since a transcranial direct current stimulation study^24^ showed that learning was enhanced with left PFC stimulation, whereas performance was negatively affected by right PFC stimulation.

For the current study, most participants started with low performance, with only two participants having an average score greater than 100 for the first day. By the last day of training, the lowest average score was 312. Day 1 performance was below the average normalized score for the junior group defined by Fried et al.,^25^ but exceeded the senior group score average by day 15. In that study, the senior group consisted of post-graduate year (PGY) 5 residents, laparoscopic surgery fellows, and practicing surgeons. Across participants, the average for the final 3 repetitions was 114 s, which is near the stated proficiency standard of 112 s.^26^ The average last day deviation of 0.56 mm is within the 1-mm limit for proficiency. In the current study, most participants needed the full 75 repetitions of training to reach these proficiency levels. This is longer than what has been reported previously. For example, Stefanidis et al.^27^ found that even for those without basic laparoscopic skill training (consistent with the current study), expert performance scores were achieved after 50±16 repetitions (310±98  min). In Vossen et al.,^28^ most participants reached the training plateau after 20 to 30 repetitions. However, the result in the current study is consistent with our previous findings on FLS intracorporeal suturing,^29^ where four participants crossed the decision threshold. Among them, they took 77, 91, and 134 repetitions to cross the threshold. Differences in the tasks, training schedules, and participant populations may have affected the learning slopes across these studies.

Although average final day performance was high, the LC-CUSUM analysis shows that not all participants reached a stable level of proficient performance. In a few cases, participants who performed in the top half on the last day (score>450, P13 and P21) did not perform well on the LC-CUSUM and did not cross the decision threshold. P13 was able to complete the task in <70  s, but had one of the weakest performances on the penalty score, having 0 penalty on only 2 of the 75 repetitions. P04, one of the participants who did cross the decision threshold, was also fast in performing the task (under 60 s) and had poor penalty score performance. The difference is that P04 remained above the criterion score for all repetitions after day 4.

At the start of the training period, cortical activation was concentrated in left regions of the dorsolateral PFC, inferior frontal gyrus, and insula. The left lateralization for this early activation is consistent with expectations, where the left hemisphere is primarily responsible for cognitive control.^30^ The dorsolateral PFC is involved in working memory and is often activated among novices as they learn psychomotor tasks since task performance often requires high levels of motor feedback and attention in the early stages of learning.^31^^,^^32^ There were six channels that showed HbO concentration changes from baseline on both day 1 and 15. These channels were above the left dorsolateral PFC, left middle frontal gyrus, left inferior frontal gyrus, right middle frontal gyrus, and right inferior frontal gyrus. Following training, there was no longer significant activation at the insula. The observed increased PFC activation from baseline is consistent with other studies that have shown elevated PFC activation for novices compared to experts.^7^^,^^9^ Although some studies of open surgery tasks, including knot tying, have shown PFC attenuation with expertise,^9^ others have found that for complex tasks, such as laparoscopic surgery tasks (such as those in the current study), attenuation of the PFC activation did not occur even after 8 h of training and high levels of performance.^15^ Even after multiple years of training (e.g., during surgery residency), PFC activation can remain above that of experts.^17^

Although the early activation was focused in areas associated with the building of perceptual models^33^ through implicit learning, with increased proficiency, trainees had increased activation in regions associated with active sensing for explicit learning where they have a knowledge of the FLS task environment.^34^ The shift from implicit to explicit intentional learning increased the activation in the right hemisphere, including significant contrasts for the right dorsolateral PFC and parietal regions. Activation of these regions on the right suggests that the novices are first building a perceptual model for the complex FLS task environment, but once they reach a trained state during implicit learning, they can use the perceptual model of the task environment for intentional learning of the FLS task and improve performance. This finding is in line with the perception–action cycle with the development of a perceptual model that has been observed for the performance of other complex motor sequence tasks,^35^ but implicit learning of the perceptual model is not often observed for simpler motor tasks, such as simple finger tapping that primarily involves the sensorimotor areas (primary sensorimotor cortices, supplementary motor area, premotor cortex, inferior parietal cortices, basal ganglia, and anterior cerebellum).^35^ In another training study, no significant differences in PFC activation were observed for trained versus untrained individuals for suture insertion or intracorporeal knot tying.^13^ However, that study only trained participants for 45 min, which may not have been sufficient to induce cortical changes.

Those who learned the task better, resulting in a higher level of final day performance, exhibited different activation patterns from the poorer performers at the end of training. Of particular note is the increased activation among good learners over bad learners for the postcentral gyrus. The fNIRS channels were approximately over Brodmann areas 1, 2, and 3 where the short-separation regression reduced the impact of strong extracerebral hemodynamic changes.^21^ Therefore, the hemodynamic activation among good learners over bad learners is in the cortical areas associated with somatosensory function, including sensory perception of the hands and arms, and for motor learning.^36^

### 4.1 Limitations and Future Work

There are a few limitations to the study that should be acknowledged. First, the amount of training may not have allowed participants to reach a true plateau of performance. Only five participants reached the decision threshold for the LC-CUSUM analysis, and others may have required additional practice to reach consistent proficient performance. In the current study, participants completed 75 repetitions over ∼6.5  h. This is consistent with the FLS curriculum guidance, which recommends 80 repetitions of practice,^19^ and prior work that found ∼8  h of training can lead to plateaued performance for laparoscopic suturing.^37^ The number of training sessions and repetitions was restricted by the implementation of the block design for effective portable neuroimaging, feasibility in maintaining participation over multiple weeks, and minimizing the development of fatigue with extended session length. Second, participants did not receive direct feedback from the researchers after each repetition. To maintain consistency of interaction across individuals and wanting brain activation to return to a resting state between repetitions for the fNIRS analysis, discussion between the researchers and participants during recording was minimized. Participants could monitor their performance based on the visual task feedback, but performance may have progressed differently if they received direct feedback on task strategy. Third, the fNIRS montage covered the prefrontal and sensorimotor areas. Other cortical regions not covered by the low-density fNIRS montage may be activated during the learning process. Future work should investigate the use of additional fNIRS channels for finer identification of relevant activation.

## Code, Data, and Materials Availability

The data that support the findings of this article are not publicly available due to IRB restrictions. They can be requested from the author at loracavu@buffalo.edu.
