# Supplementary material for: Changes in functional neuroimaging measures as novices gain proficiency on the fundamentals of laparoscopic surgery suturing task
Source: Neurophotonics. 2023 May 5;10(2):023521. doi: 10.1117/1.NPh.10.2.023521 (PMC10160767; doi:10.1117/1.NPh.10.2.023521)
Supplement: Supplementary file 1 [file NPh_010_023521_SD001.pdf]

Supplementary Materials

Table S1. p values for the comparison of cortical activation for Day 1 and Day 15. * indicates a significant difference either from baseline or for the day-to-day contrast.

Source	Detector	HbO Day 1	HbR Day 1	HbO Day 15	HbR Day 15	HbO Day 1 vs. Day 15	HbR Day 1 vs. Day 15
1	1	0.046*	0.127	0.001*	0.151	0.509	0.621
1	15	0.209	0.211	0.059	0.314	0.826	0.474
2	2	0.363	0.154	0.081	0.281	0.116	0.960
2	3	0.633	0.102	0.786	0.878	0.899	0.322
3	1	0.013*	0.452	<0.001*	0.196	0.151	0.800
3	3	0.015*	0.062	0.367	0.855	0.442	0.253
3	5	0.018*	0.194	0.002*	0.147	0.844	0.548
4	1	0.047*	0.944	0.000*	0.878	0.080	0.971
4	3	0.072	0.338	0.589	0.131	0.557	0.644
4	15	0.748	0.256	0.360	0.575	0.726	0.273
5	2	0.945	0.613	0.348	0.636	0.331	0.875
5	3	0.988	0.865	0.973	0.042*	0.987	0.176
5	4	0.531	0.026*	0.938	0.981	0.572	0.111
5	5	0.903	0.990	0.211	0.674	0.255	0.781
6	4	0.002*	0.413	0.051	0.669	0.230	0.342
6	5	0.136	0.627	0.010*	0.888	0.954	0.793
6	6	0.550	0.108	0.403	0.978	0.986	0.162
7	2	0.305	0.518	0.649	0.638	0.647	0.289
7	4	0.399	0.109	0.066	0.681	0.438	0.191
7	7	0.673	0.060	0.801	0.480	0.506	0.066
8	4	0.745	0.390	0.070	0.245	0.308	0.908
8	6	0.254	0.001*	0.759	0.314	0.544	0.039*
8	7	0.546	0.191	0.138	0.334	0.426	0.109
9	8	0.066	0.825	0.000*	0.501	0.125	0.532
9	15	0.191	0.084	0.027*	0.518	0.970	0.238
10	8	0.057	0.222	0.003*	0.050*	0.209	0.989
10	9	0.339	0.358	0.015*	0.590	0.130	0.701
10	13	0.028*	0.389	0.019*	0.192	0.624	0.153
11	9	0.414	0.315	0.347	0.357	0.832	0.994
11	10	0.649	0.305	0.991	0.117	0.750	0.582
12	8	0.223	0.188	0.001*	0.063	0.205	0.473
12	9	0.543	0.314	0.009*	0.312	0.166	0.983
12	15	0.142	0.411	0.221	0.539	0.845	0.808
13	9	0.638	0.024*	0.283	0.034*	0.782	0.646
13	10	0.364	0.877	0.905	0.119	0.476	0.231
13	12	0.154	0.038*	0.026*	0.462	0.010*	0.277
13	13	0.508	0.585	0.092	0.849	0.536	0.665
14	12	0.663	0.236	0.187	0.035	0.680	0.883
14	13	0.013*	0.113	0.150	0.006*	0.418	0.642
14	14	0.810	0.073	0.029*	0.182	0.069	0.721
15	10	0.405	0.230	0.864	0.687	0.433	0.164
15	11	0.419	0.223	0.084	0.795	0.049*	0.489
15	12	0.134	0.022*	0.181	0.049*	0.714	0.396
16	11	0.290	0.939	0.446	0.625	0.129	0.616
16	12	0.325	0.020*	0.565	0.099	0.703	0.248
16	14	0.711	0.003*	0.753	0.001*	0.619	0.150

Table S2. p values for the comparison of cortical activation on Day 15 for high performing versus low performing group. * indicates a significant difference for the group contrast.

**Source**	**Detector**	**HbO Day 15**	**HbR Day 15**
1	1	0.936	0.174
1	15	0.430	0.220
2	2	0.072	0.512
2	3	0.032*	0.131
3	1	0.875	0.582
3	3	0.005*	0.386
3	5	0.886	0.152
4	1	0.836	0.215
4	3	0.004*	0.080
4	15	0.307	0.498
5	2	0.195	0.505
5	3	0.205	0.839
5	4	0.694	0.803
5	5	0.006*	0.583
6	4	0.565	0.112
6	5	0.293	0.132
6	6	0.791	0.394
7	2	0.309	0.840
7	4	0.511	0.632
7	7	0.916	0.880
8	4	0.011*	0.963
8	6	0.642	0.682
8	7	0.712	0.443
9	8	0.209	0.685
9	15	0.260	0.128
10	8	0.102	0.329
10	9	0.067	0.252
10	13	0.245	0.617
11	9	0.896	0.416
11	10	0.446	0.412
12	8	0.339	0.727
12	9	0.887	0.821
12	15	0.121	0.320
13	9	0.084	0.769
13	10	0.371	0.194
13	12	0.047*	0.195
13	13	0.018*	0.940
14	12	0.268	0.646
14	13	0.086	0.994
14	14	0.630	0.843
15	10	0.966	0.261
15	11	0.050*	0.907
15	12	0.138	0.230
16	11	0.034*	0.503
16	12	0.217	0.820
16	14	0.261	0.257
